# Eye Movement Impairment in Women Undergoing Chemotherapy

**DOI:** 10.3390/jemr18050041

**Published:** 2025-09-11

**Authors:** Milena Edite Casé de Oliveira, José Marcos Nascimento de Sousa, Gerlane Da Silva Vieira Torres, Ruanna Priscila Silva de Brito, Nathalia dos Santos Negreiros, Bianca da Nóbrega Tomaz Trombetta, Kedma Anne Lima Gomes Alexandrino, Waleska Fernanda Souto Nóbrega, Letícia Lorena Soares Silva Polimeni, Catarina Cavalcanti Braga, Cristiane Maria Silva de Souza Lima, Thiago P. Fernandes, Natanael Antonio dos Santos

**Affiliations:** 1 Department of Psychology, Centro Universitário Tabosa de Almeida, Caruaru 55016-400, Pernambuco, Brazil; 2023243198@app.asces.edu.br (L.L.S.S.P.); 2021120243@app.asces.edu.br (C.C.B.); 2023243192@app.asces.edu.br (C.M.S.d.S.L.); 2 Department of Psychology, Universidade Federal da Paraíba, João Pessoa 58051-900, Paraíba, Brazil; jmns@academico.ufpb.br (J.M.N.d.S.); gerlanetorressv@gmail.com (G.D.S.V.T.); ruanna.priscila@academico.ufpb.br (R.P.S.d.B.); psinathalianegreiros@gmail.com (N.d.S.N.); biancanobregatt@gmail.com (B.d.N.T.T.); thiagompfernandes@gmail.com (T.P.F.); natanael_labv@yahoo.com.br (N.A.d.S.); 3 Department of Physiotherapy, Universidade Estadual da Paraíba, Campina Grande 58432-300, Paraiba, Brazil; kedma.gomes@maisunifacisa.com.br; 4 Department of Dentistry, Universidade Federal de Minas Gerais, Belo Horizonte 31270-901, Minas Gerais, Brazil

**Keywords:** breast cancer, chemotherapy, eye tracker, cognition, neuroscience

## Abstract

The assessment of visual attention is important in visual and cognitive neuroscience, providing objective measures for researchers and clinicians. This study investigated the effects of chemotherapy on eye movements in women with breast cancer. Twelve women with breast cancer and twelve healthy controls aged between 33 and 59 years completed a visual search task, identifying an Arabic number among 79 alphabetic letters. Test duration, fixation duration, total fixation duration, and total visit duration were recorded. Compared to healthy controls, women with breast cancer exhibited significantly longer mean fixation duration [t = 4.54, *p* < 0.00]; mean total fixation duration [t = 2.41, *p* < 0.02]; mean total visitation duration [t = 2.05, *p* < 0.05]; and total test time [t = 2.32, *p* < 0.03]. Additionally, positive correlations were observed between the number of chemotherapy cycles and the eye tracking parameters. These results suggest the possibility of slower information processing in women experiencing acute effects of chemotherapy. However, further studies are needed to clarify this relationship.

## 1. Introduction

Female breast cancer (BC) is the most diagnosed neoplasm in the world, with an estimated 2.3 million new cases for the year 2020 [1]. Chemotherapy is one of the main treatments for BC and has contributed substantially to improving in this affected population [2]. However, chemotherapy can affect several regions of the central nervous system and may impair visual processing and cognitive functions [3]. In this regard, chemotherapy-related cognitive impairment (CRCI), or “chemobrain”, has been widely reported in the literature [4]. Changes in attention, verbal fluency, verbal memory, and visual processing have been reported after chemotherapy treatment, which can have a significant impact on the quality of life and work capacity in this population [5].

Cognition is complex and depends on several important neural structures [6]. Measuring cognitive function in patients undergoing a pharmaceutical intervention or neurological disease can help investigate the impact these conditions can have on individuals’ daily lives [7]. In healthy individuals, cognitive changes can be predictive of accident rates, for example [8]. Several techniques have been implemented to objectively assess cognitive function, such as the neuropsychological test battery [9,10,11]. However, to date, no study has used an eye-tracking technique to assess cognitive function in women with BC.

Eye movements offer valuable insights into how cognition affects human behavior and perception [12]. In this regard, the relationship between eye-tracking parameters and cognitive domains is being widely considered in scientific studies [13]. As eye-tracking is considered an accessible, non-invasive and easy-to-apply technique, the identification of eye movement parameters can be a useful measure for the development of investigative protocols in diverse populations [14].

Thus, the objective of the present study was to assess whether eye movement patterns, such as fixation and visit durations, are altered in women with BC undergoing chemotherapy. Our hypothesis was that the group of healthy women would perform the visual search task more efficiently than the women in the clinical group.

## 2. Materials and Methods

### 2.1. Study Design

Quantitative, quasi-experimental, analytical, cross-sectional study conducted between April 2021 and May 2023.

### 2.2. Ethical Statements

This study adhered to the principles of the Declaration of Helsinki for research involving human subjects and was approved by the Ethics Committee of a Brazilian Public University (CAAE: 42983821.6.0000.5188). Participation in the research was voluntary, and all participants signed an informed consent form to participate in the research.

### 2.3. Participants and Eligibility Criteria

The study consisted of 12 women with BC undergoing chemotherapy treatment (CT) (mean age = 47.17 years, SD = 6.79 years) and 12 healthy controls (HC) (mean age = 42.92 years, SD = 6.00 years). Participants were recruited from a reference hospital for the treatment of BC in Northeast Brazil, or through dissemination in digital media (radio, flyers, Instagram). The groups were matched by age and education.

Women aged 33–59 years, with normal or corrected-to-normal vision (at least 20/20) as assessed by a Snellen chart were included [15]. Participants were tested at 60 cm. To mitigate the effects of presbyopia, all participants used correction when necessary (e.g., multifocal lenses, or reading glasses for participants who wore lenses). Acuity was verified with standardized optotypes, and only then was the eye tracker calibrated and validated (i.e., 9-point fixation test). In addition, an initial test with identical stimuli confirmed ≥ 80% performance before the main task. The inclusion criterion for CT was to have finished chemotherapy treatment against BC about five days before the start of the evaluation.

The exclusion criteria adopted for the study groups were as follows: current history of neurological conditions, chronic cardiovascular diseases, brain damage, previous exposure to neurotoxic agents, current or previous abuse of psychoactive substances, continuous use of drugs with a potential effect on visual processing and cognition, and comorbidities such as diabetes mellitus, hypertension, HIV, schizophrenia, bipolar disorder were excluded. In addition, women with any cognitive deficit or other ocular comorbidity that could affect eye movements or visual acuity were excluded [16].

Exclusion criteria for the CT were: (a) disease recurrence; (b) having undergone another antineoplastic treatment; (c) having been diagnosed with cancer in the past. The HCs were recruited from the general population and the participants reported not having any of the exclusion criteria adopted.

### 2.4. Procedure and Stimulus

The experiment was conducted in a silent room with standardized ambient lighting using diffused white, fluorescent lamps, distributed on the ceiling, without direct incidence on the monitor or the participants’ eyes. The average luminance of the monitor (23”, 1920 × 1080 px, 300 cd/m^2^), was set according to eye-tracking protocol recommendations. Luminance was assessed using a ColorCal MKII (Cambridge Research Systems, Kent, London).

**Figure 1:**
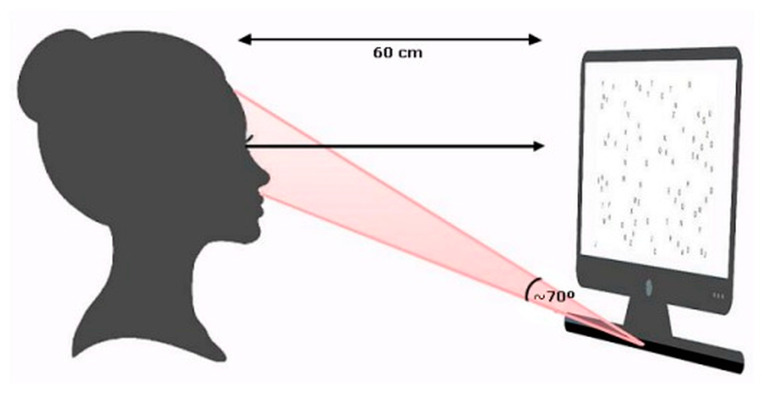
Representation of eye-tracking equipment.

Participants were positioned approximately 60 cm from the screen, using a height-adjustable chair to ensure eye alignment with the center of the monitor and ergonomic standardization. An eye tracker with a 300 Hz sampling frequency was used, coupled to the monitor and operated by Tobii Studio software (v. 3.4.0), Tobii TX300 Eye Tracker Model, which enabled programming, stimulus presentation, data recording, and descriptive analysis of eye movements (Figure 1).

Given the high precision and motion tolerance of the Tobii system, no head stabilizer was used to maintain natural visual conditions and minimize interference with oculomotor behavior. All tests were conducted in binocular vision, with normal or corrected visual acuity.

Calibration was performed individually using the software’s five-point protocol and repeated until the calibration parameters were correct. The required precision was ≤0.5° and accuracy ≤ 0.3°. The procedure was repeated up to three times as needed, using visual feedback from the software for adjustments. All participants met the criteria, and no exclusions were required.

Between stimulus presentations, a central fixation target was displayed for three seconds. Before the start of the experiment, participants received detailed instructions on the experimental procedures.

### 2.5. Visual Search Task

Participants received verbal instructions, followed by five training templates prior to task onset. Participants were asked to fixate their gaze on the central target (+) to ensure a stationary fixation location at the beginning of each new trial. The “enter” key on the keyboard was used to initiate the test phase.

**Figure 2:**
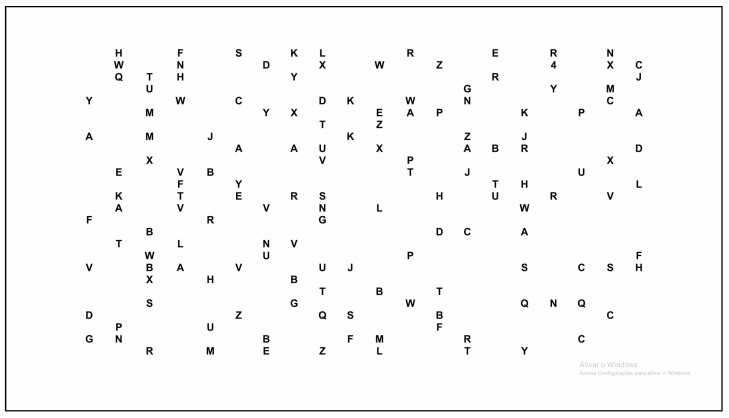
Display of the visual lookup table with 79 letters of the alphabet and one number.

The visual search task consisted of presenting a single number among 79 letters of the alphabet, randomly distributed on the screen. The letters “I” and “O” were excluded due to visual ambiguity [13]. The Arabic numerals used were 4, 6, 7 and 9, as they are considered more easily discriminated numbers from the alphabet [17]. Each stimulus was delimited by a rectangular Area of Interest (AOI), with a standardized size equivalent to 0.85° of visual angle, avoiding overlap. A total of 80 AOIs were generated per trial. The positions of the stimuli were defined by a random algorithm, with a restriction against repeated groupings in the same quadrant. The rectangular format was adopted because it is compatible with Tobii Studio’s fixation algorithm, which considers fixations ≥200 ms within the AOI. For further analysis, the entire stimulus matrix was considered a global AOI (Figure 2).

The size of the fixing cross, alphabets and numbers were defined with a visual angle of 0.85° [13]. The location of the target number was randomized on each trial, with the rule of not being in the same visual quadrant for more than three successive trials. Participants were instructed to search for the number as quickly as possible and then report aloud when the number was located. Then, the applicator presented the next task to be performed. The visual search task had 37 trials after the five practice runs.

The eye movement parameters assessed included test duration, fixation duration, total fixation duration, and total visit duration. Test duration corresponds to the total time taken by the participant to complete the visual task. Fixation duration measures the time when the eyes are relatively fixed, assimilating or decoding the information presented. Total fixation duration measures the average duration of fixations within the areas of interest throughout the test. Fixations were defined by Tobii Studio’s default fixation algorithm as steady gazes with positions remaining for at least 200 ms. Total visit duration measures the duration of all visits made within the areas of interest, including the number of fixations and saccades.

Quality of life was assessed using the Medical Outcomes Study 36-Item Short-Form Health Survey (SF-36). This is a self-report questionnaire made up of 36 items divided into eight domains: Functional Capacity (10 items), Physical Limitations (4 items), Pain (2 items), General Health Status (5 items), Vitality (4 items), Social Aspects (2 items), Emotional Limitations (3 items) and Mental Health (5 items). The scores for each domain are transformed into a scale from 0 to 100, where higher values indicate a better perception of health and quality of life.

### 2.6. Statistical Analysis

Statistical analyses were performed using SPSS version 20. The Shapiro–Wilk test showed that the data met the assumption of normality; therefore, parametric tests were applied. Comparisons of sociodemographic and clinical measures were performed using Student’s *t*-test. Pearson’s correlation tests were used to assess the relationships between eye movement parameters, the number of chemotherapy cycles, and quality of life domains. A *p*-value of <0.05 was considered statistically significant.

## 3. Results

The sample included 24 participants (12 CT and 12 HC), aged between 33 and 59 years. The groups did not differ with respect to age [t (22) = 1.623, *p* = 0.119]; education [t (22) = −0.235, *p* = 0.816]; depression scores [t (22) = 0.453, *p* = 0.655]; or anxiety [t (22) = 0.906, *p* = 0.375].

Regarding the clinical features of breast cancer, all women were diagnosed with invasive ductal carcinoma. Regarding histologic grade, two (17%) women had grade III, while ten (83%) were classified with grade II. The ki-67 protein (cell proliferation marker) showed a variation between 10% and 60%. In this regard, seven (58%) women had ki-67 10%, followed by two (16%) ki-67 at 15%; two (16%) ki-67 at 20% and one (10%) with ki-67 at 60%. The drugs used by participants with or without interaction were as follows: cyclophosphamide, doxorubicin, carboplatin, paclitaxel, docetaxel, genuxal and trastuzumab.

Regarding the domains of quality of life, the CT presented lower scores for: (i) functional capacity [t (22) = −4.907, *p* = 0.00, g = −1.93, 95% CI = −2.88 to −0.99]; (ii) physical limitation [t (22) = −4.962, *p* = 0.00, g = −1.96, 95% CI = −2.91 to −1.01]; and (iii) social aspects [t (22) = −2.514, *p* = 0.02, g = −0.99, 95% CI = −1.81 to −0.17], when compared to the control group (Table 1). In addition, the correlation tests performed suggested a negative relationship between the number of chemotherapy cycles and the quality of life domains: (i) functional capacity [r = −0.72, *p* = 0.00]; (ii) physical limitation [r = −0.56, *p* = 0.00]; and (iii) social aspects [r = −0.42, *p* = 0.03].

**Figure 3:**
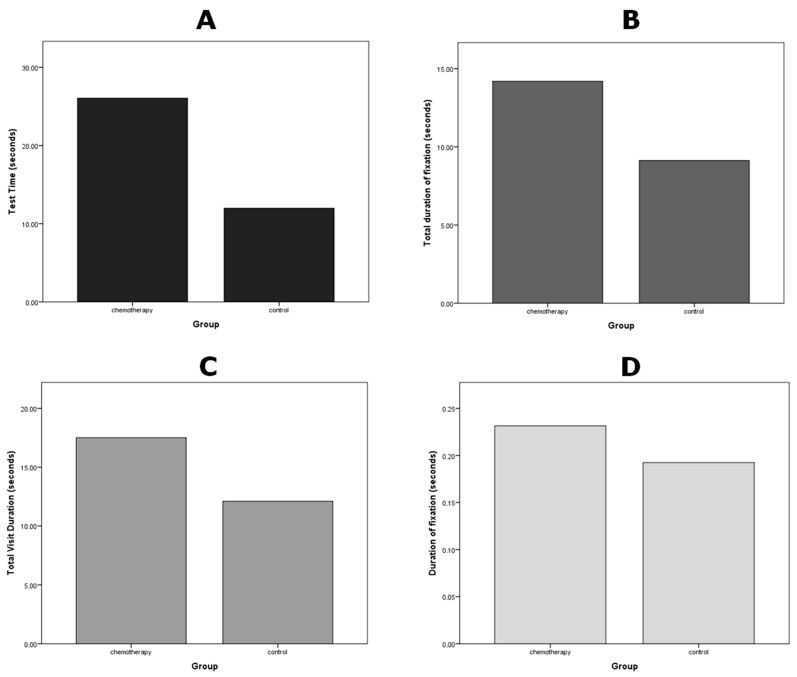
Bar graphs representing the means of eye movement parameters in the visual search task per group, where (**A**) = Total Test Time (seconds) (t = 2.32, *p* = 0.03 *); (**B**) = Total Fixation Duration per area of interest (seconds) (t = 2.41, *p* = 0.02 *); (**C**) = Total Visit Duration per area of interest (seconds) (t = 2.05, *p* = 0.05 *); (**D**) = Fixation Duration per area of interest (t = 4.54, *p* = 0.00 *, Mean ± SD: Chemotherapy = 0.23 ± 0.20, Control = 0.19 ± 0.21). * Significant results.

Main effects of study group on eye movement parameters were analyzed using independent samples *t*-tests. The results indicated significant difference for the four outcomes of interest: (i) mean fixation duration [t (22) = 4.54, *p* < 0.00 *, g = 0.8, 95% CI = 0.84 to 2.01]; (ii) mean total fixation duration [t (22) = 2.41, *p* = 0.02 *, g = 0.95, 95% CI = 0.14 to 1.77]; (iii) mean total visit duration [t (22) = 2.05, *p* = 0.05 *, g = 0.81, 95% CI = 0.00 to 1.61]; and (iv) total test time [t (22) = 2.32, *p* = 0.03 *, g = 0.92, 95% CI = 0.10 to 1.73] (Table 1) (Figure 3).

Correlation tests were performed to assess the relationship between eye tracking parameters and participants’ clinical variables: number of chemotherapy cycles and quality of life domains. The results indicated a positive relationship between the number of chemotherapy cycles and all eye-tracking parameters evaluated, namely: (i) fixation duration [r = 0.68, *p* < 0.001]; (ii) total fixation duration [r = 0.49, *p* = 0.01]; (iii) total visit duration [r = 0.50, *p* = 0.01]; and (iv) total test time [r = 0.51, *p* = 0.01] (Table 2).

Regarding the relationship with the quality of life domains, significant negative correlations were observed between (i) duration of fixation and functional capacity [r = −0.48, *p* = 0.01] and physical limitation [r = −0.60, *p* = 0.00]; (ii) total duration of fixation and functional capacity [r = −0.56, *p* = 0.00]; (iii) total duration of visit and functional capacity [r = −0.56, *p* = 0.00]; and (iv) total test time and functional capacity [r = −0.48, *p* = 0.01] and physical limitation [r = −0.47, *p* = 0.01] (Table 2).

## 4. Discussion

Our study sought to assess whether fixation and visitation time can be altered in women with BC undergoing chemotherapy treatment. Our results support the hypothesis that the acute effect of chemotherapy may lead to alteration in eye movement patterns, through the application of a visual search task validated as an indicator of cognitive process in patients with neurological disorders [13]. This is the first study to assess this relationship in patients with BC under the acute effect of chemotherapy.

Evidence that aids in the individual’s decision making is accumulated during the fixation process. Thus, attention is considered to play an active role in the choice of visual targets [18]. It is during fixations that we plan orientation and direct gaze, suggesting the existence of cognitive processing during action [19].

Considering that in the present study the two groups evaluated presented a 100% hit rate in the identification of the target of interest, the longer fixation and visitation time may be indicative of greater complexity for stimulus recognition. As the CT generated longer fixation time on the target of interest as well as longer total test time, it is suggested that participants required more effort to process the information, providing an explanation of how impaired cognitive processing limits the daily functioning of women with BC. In this regard, found negative correlations between mean fixation time and verbal performance, pattern recognition memory, and verbal fluency test in Parkinson’s patients [13].

As chemotherapy has been associated with other measures of visual processing [11,20,21], it was expected that changes in eye-tracking would also be observed. This investigation is justified by the fact that the retina derives from the same embryonic layer as the central nervous system and is a neural extension directly connected to the optic nerve. Consequently, visual alterations can reflect dysfunctions in brain activity [22].

Studies have suggested that individuals with impairment in visual regions, such as the retina or optic nerve, may experience difficulties in reading and identifying visual patterns [23]. Because longer fixations correlate negatively with visual search efficiency, eye movements are closely associated with cognitive processing [24]. The hypothesis is that this is because the individual is spending more time interpreting or relating the representations of the components presented in the visual task to internalized representations [24].

Evidence that chemotherapy affects cognitive domains such as verbal fluency and visual memory has already been presented in the literature [25,26]. Semantic memory and sustained attention are also impaired after chemotherapy administration [27]. Evidence indicates that the adverse effects of chemotherapy on cognition are associated with biochemical and morphological changes in the brain, such as the reduction in microglial cells in the prefrontal cortex [28]. Studies in animal models have shown that doxorubicin induces an imbalance between reactive oxygen species and antioxidant enzymes, resulting in structural damage to the hippocampus [29]. For humans, the literature also describes a reduction in gray and white matter volumes after chemotherapy treatment [30,31].

The mechanism of action of chemotherapeutic drugs on the nervous system can occur through different pathways. Chemotherapy-induced peripheral neuropathy, especially paclitaxel and docetaxel, is characterized by damage to nerves that control movement and sensory processing [32]. Cyclophosphamide and fluorouracil can cross the blood–brain barrier and trigger cell death [33,34]. Doxorubicin and taxanes can trigger damage to the nervous system indirectly, through the inflammatory process that is promoted by their mechanism of action [32]. Furthermore, chemotherapy was associated with driving sustained inflammatory processes, cytokine downregulation and induction of monocyte migration into the Central Nervous System [35,36]. Such changes can trigger damage to visual functions, including eye movement control, as well as other neural structures related to cognitive processes such as attention, inhibitory control, reaction time and decision making [32].

Although it is not easy to separate the effects of diagnosis, BC and chemotherapy treatment, the results of our study suggested a positive correlation between the amount of chemotherapy cycles administered and the eye movement parameters assessed. In this regard, previous evidence suggests that neurotoxic effects from chemotherapy depend on the total cumulative dose administered [37].

Our results also suggested a negative correlation between quality of life domains: functional capacity, physical limitation, and social aspects and the number of chemotherapy cycles administered. Negative effects such as physical discomfort, nausea and social isolation resulting from chemotherapy treatment have been reported in the literature, which may affect the quality of life of women undergoing this treatment [38,39].

Eye movement parameters were also negatively associated with functional capacity and physical limitation. Thus, the shorter the fixation times and visit durations, the higher the scores in the these quality of life domains. Growing evidence suggests that decision making and information processing are intrinsically linked, and impairments in these parameters can negatively impact work and daily activities, leading to loss of social autonomy in clinical populations [40].

This study has some limitations. Although we controlled for potential confounding variables matching the groups for age, education, and anxiety and depression scores, other clinical variables may influence on the results found, such as cancer itself. However, this is a factor inherent to our study design and therefore could only be addressed in preclinical models. While environmental variables were controlled, the absence of quantitative measures of continuous visual engagement is a limitation. We recommend that future studies incorporate these analyses to enhance robustness. Additionally, the lack of formal assessment of fatigue or sleep quality may have influenced ocular parameters, especially in the chemotherapy group, where these symptoms are prevalent. Future studies could include other validated scales, such as the Pittsburgh Sleep Quality Index (PSQI) or the Fatigue Severity Scale (FSS), to control these variables.

Considering that our results indicated changes in ocular patterns associated with chemotherapy, it is important to highlight their practical implications. Parameters like prolonged fixation time could be integrated into screening protocols in oncology outpatient clinics to identify patients in need of early intervention. Future studies should focus on validating these findings in larger samples and on developing automated tools to support clinical implementation.

The results found in this study indicate that alterations in oculomotor patterns, such as a significant increase in fixation time, are associated with significant functional impairments, especially in tasks that require sustained visual attention. This evidence reinforces the importance of incorporating visual assessments into the clinical routine of patients undergoing chemotherapy treatment, with a view to the early identification of subtle cognitive impairments. In addition, the findings support the proposal of practical measures, such as adaptations to the working day for intensive visual tasks, individualized advice on driving and the development of assistive technologies based on eye-tracking. These measures have the potential to mitigate the functional impacts of chemotherapy and should be considered in the formulation of clinical guidelines and public policies aimed at preserving the quality of life of these patients.

Although it provides relevant evidence on the effects of chemotherapy on visual processing, the small number of participants makes it difficult to generalize the results. This limitation was due to challenges in recruiting the sample, such as treatment-related fatigue and strict exclusion criteria, which impacted on patient eligibility and availability. Even so, the study provides initial data on a population that is difficult to access and poorly represented in the literature. Future research should consider strategies such as multicenter recruitment and longitudinal designs, with the aim of expanding the sample, strengthening external validity and deepening the understanding of the cognitive effects of chemotherapy. Furthermore, although the study focused on the acute effects of chemotherapy, longitudinal studies are needed to determine whether these ocular alterations persist, normalize or evolve differently after months of chemotherapy.

Despite differences in the drugs used and the number of cycles administered in each participant, they all received chemotherapeutic agents with a similar mechanism of action. It is important to consider that our results assessed the acute effect of chemotherapy on the body of women with BC. In this regard, longitudinal studies could help in understanding how these effects present themselves after the treatment remission period.

## 5. Conclusions

Women with BC undergoing chemotherapy regimens have altered eye movement patterns, and we believe that this may be due to the toxicity triggered in the visual system. These results may have direct implications for basic and clinical research, assisting in the development of care plans for this population. Further investigations into the relationship between chemotherapy and visual processing are needed, implementing additional investigative techniques and study designs to understand how these results behave under different conditions. Thus, we hope that longitudinal and controlled studies can be conducted in order to better elucidate these results.

## Figures and Tables

**Table 1:** Sociodemographic and clinical characteristics and eye-tracking parameters of the sample.

	Chemotherapy (N = 12)	Control (N = 12)	
Variables	Mean ± SD	Mean ± SD	*p* Value *
Age, years	47.17 ± 6.79	42.92 ± 6.00	0.11
Chemotherapy cycles	20.58 ± 14.14	-	-
QL- Functional capacity	43.75 ± 24.22	82.50 ± 12.70	0.00 *
QL-Physical limitation	6.25 ± 11.30	60.42 ± 36.08	0.00 *
QL-Pain	46.75 ± 18.60	59.83 ± 24.28	0.15
QL-General health status	49.75 ± 19.58	59.75 ± 20.35	0.23
QL-Vitality	48.75 ± 17.98	54.58 ± 10.54	0.34
QL-Social aspects	50.00 ± 21.32	74.00 ± 25.28	0.02 *
QL-Emotional limitation	25.96 ± 39.90	57.08 ± 41.88	0.07
QL-Mental health	58.67 ± 25.60	70.67 ± 13.02	0.16
Duration of application	26.06 ± 20.25	11.97 ± 5.43	0.03 *
Duration of fixation (seconds)	0.23 ± 0.20	0.19 ± 0.21	0.00 *
Total duration of fixation (seconds)	14.18 ± 6.38	9.12 ± 3.43	0.02 *
Total visit duration (seconds)	17.52 ± 7.63	12.10 ± 5.07	0.05 *

QL = Quality of life. Note: SF-36 scores range from 0 to 100, with higher values indicating better health and functioning. * Significant results.

**Table 2:** Correlation tests performed between eye tracking parameters and clinical characteristics of the study groups.

	Test Duration		Duration of Fixation		Total Duration of Fixation		Total Visit Duration	
Variables	*r*	*p* Value	*r*	*p* Value	r	*p* Value	r	*p* Value
Chemotherapy cycles	0.51	0.01 *	0.68	0.00 *	0.49	0.01 *	0.50	0.01 *
QL–Functional capacity	−0.48	0.01 *	−0.48	0.01 *	−0.56	0.00 *	−0.56	0.00 *
QL–Physical limitation	−0.47	0.01 *	−0.60	0.00 *	−0.31	0.13	−0.19	0.36

* QL—Quality of Life.

## Data Availability

The data can be provided upon request.

## Abbreviations

The following abbreviations are used in this manuscript:

BC	Breast Cancer
CRCI	Chemotherapy-related Cognitive Impairment
CT	Chemotherapy Treatment
HC	Healthy Controls
QL	Quality of Life
